# Fidelity of DNA replication—a matter of proofreading

**DOI:** 10.1007/s00294-018-0820-1

**Published:** 2018-03-02

**Authors:** Anna Bębenek, Izabela Ziuzia-Graczyk

**Affiliations:** Institute of Biochemistry and Biophysics, Polish Academy of Sciences, Pawińskiego 5a, 02-106 Warsaw, Poland

**Keywords:** Replicative polymerases, 3′-5′ proofreading, Polymerase structure, Fidelity

## Abstract

DNA that is transmitted to daughter cells must be accurately duplicated to maintain genetic integrity and to promote genetic continuity. A major function of replicative DNA polymerases is to replicate DNA with the very high accuracy. The fidelity of DNA replication relies on nucleotide selectivity of replicative DNA polymerase, exonucleolytic proofreading, and postreplicative DNA mismatch repair (MMR). Proofreading activity that assists most of the replicative polymerases is responsible for removal of incorrectly incorporated nucleotides from the primer terminus before further primer extension. It is estimated that proofreading improves the fidelity by a 2–3 orders of magnitude. The primer with the incorrect terminal nucleotide has to be moved to exonuclease active site, and after removal of the wrong nucleotide must be transferred back to polymerase active site. The mechanism that allows the transfer of the primer between pol and exo site is not well understood. While defects in MMR are well known to be linked with increased cancer incidence only recently, the replicative polymerases that have alterations in the exonuclease domain have been associated with some sporadic and hereditary human cancers. In this review, we would like to emphasize the importance of proofreading (3′-5′ exonuclease activity) in the fidelity of DNA replication and to highlight what is known about switching from polymerase to exonuclease active site.

## Introduction

Maintaining a low mutation rate is essential for cell viability and health. It was estimated that both in prokaryotic and eukaryotic cells, DNA is replicated with the very high fidelity with one wrong nucleotide incorporated once per 10^8^–10^10^ nucleotides polymerized. The accuracy of replication relies heavily on the ability of replicative DNA polymerases to efficiently select correct nucleotides for the polymerization reaction and excise mistakenly incorporated nucleotides using their intrinsic exonucleases. DNA replication is constantly challenged by endogenous and exogenous chemicals, non-canonical DNA structures, and difficult to replicate DNA sequences. Both prokaryotic and eukaryotic cells possess a variety of specialized DNA polymerases that help to overcome replication blocks and that are subsequently recruited to DNA by PCNA modifications (Li et al. 2017; Zhao and Washington 2017).

Eukaryotic cells are known to contain at least 16 different DNA polymerases while prokaryotic cells like an *Escherichia coli* have five different DNA polymerases (Goodman and Tippin 2000; Kunkel 2009). All known polymerases are divided into six families, A, B, C, D, X, and Y, on the basis of their sequence conservation (Bebenek and Kunkel 2004; Kunkel 2009). Recently, 17 human DNA polymerases have been purified and biochemically characterized an AEP (archaeo-eukaryotic primase) superfamily (Rudd et al. 2014).

Genomic DNA replication is normally carried out by the polymerases from A, B, C, or D families with high fidelity and processivity. Others, known as specialized, bypass, or translesion polymerases, participate in various DNA transactions related to repair, genome stability, and the generation of antibody diversity (Kunkel 2009; Shcherbakova et al. 2003a; Sweasy et al. 2006; Zhao and Washington 2017).

Replicative polymerases are also present in bacteriophages. The best characterized are B family polymerases from bacteriophages T4 and RB69, and A family polymerase from T7 bacteriophage (Johnson 2010; Karam and Konigsberg 2000).

In eukaryotic cells, replication of genomic DNA requires minimally three DNA polymerases, polymerase α, δ, ɛ all from B family (Burgers and Kunkel 2017; Johansson and Macneill 2010; Makarova and Burgers 2015; Pellegrini 2012; Tahirov 2012), and polymerase γ from A family that is responsible for mitochondrial DNA replication (Copeland 2010). The fourth polymerase that operates at replication fork is polymerase ζ also from B family, but this polymerase does not carry a bulk of DNA replication. Its role is critical at the difficult templates sites or when replicative DNA pols are compromised. Four subunits Pol ζ is less accurate than other members of B family and does not have a proofreading activity (Makarova and Burgers 2015; Szwajczak et al. 2017). In the *E. coli*, genomic DNA replication is carried out by polymerase III (C family), Pol II (B family), and Pol I (A family) and the archaea genomes are replicated by the polymerases from D and B families (Banach-Orlowska et al. 2005; Cann and Ishino 1999; Edgell and Doolittle 1997; Kornberg and Baker 1992).

The replicative polymerase of *E. coli*, DNA polymerase III, is a ten subunit complex that is categorized into three major components; the Pol III core, the β-clamp, and the γ-complex. The Pol III core is a heterotrimer composed of the α polymerase, ɛ 3′-5′ proofreading exonuclease, and θ subunit of an unknown function (Johnson and O’Donnell 2005). The eukaryotic replicative polymerases are also multiple subunits holoenzymes. Polymerase α is comprised of a catalytic subunit p180 and an accessory subunit p70, and is a part of a four subunit pol-prim DNA primosome (Muzi-Falconi et al. 2003). The human DNA pol δ is heterotetrameric complex, consisting of the catalytic subunit p125 (POLD1) and three accessory subunits p50 (POLD2), p68 (POLD3), and p12 (POLD4) (Tahirov 2012). The *Saccharomyces cerevisiae* pol δ is a three-subunit complex consisting of a catalytic Pol3p subunit and two accessory subunits Pol31p and Pol32p subunit (Tahirov 2012). DNA pol ɛ is composed of the catalytic subunit Pol2 (POLE) and three non-catalytic subunits Dpb2 (POLE2), Dpb3 (POLE3), and Dpb4 (POLE4) (Hogg and Johansson 2012).

## Replication fidelity

Replicative polymerases achieve high fidelity of DNA replication by employing several mechanisms: (1) sensing proper geometry of correct base pair, (2) slowing down catalysis in case of a mismatch, and (3) partitioning the mismatched primer to exonuclease active site.

Polymerase selectivity is the prime determinant of fidelity. It is estimated that polymerases make errors approximately once every 10^4^–10^5^ nucleotide polymerized (Echols and Goodman 1991; Showalter and Tsai 2002). Proofreading enhances the overall fidelity of DNA synthesis by a factor 10^2^–10^3^ depending on the specific DNA polymerase and the nature of the primer terminal mispair (Kunkel 2009; Kunkel and Burgers 2008; McCulloch and Kunkel 2008; St Charles et al. 2015).

Structural studies of replicative DNA polymerase families showed that polymerase (pol) and 3′-5′ exonuclease (exo) activities reside on one polypeptide. The exceptions are eukaryotic polymerases α and ζ, that do not have functional exonuclease activity (Abbotts and Loeb 1985; Makarova and Burgers 2015), and polymerase III from *E. coli*, where exonuclease activity is carried out on different subunit (ɛ subunit) encoded by a *dnaQ* gene (Maki and Kornberg 1987; Scheuermann and Echols 1984).

### B- family polymerase structures

T4 DNA polymerase, a product of phage gene *gp43*, was the most intensely studied polymerase from B family and for many years served as a key model of replicative polymerase (Karam and Konigsberg 2000). The intact T4 DNA polymerase has never been crystallized, but fortunately, the polymerase of the related bacteriophage RB69 in apo conformation was crystallized by Steitz group in 1997 (Wang et al. 1997). Subsequently, the structures of ternary complexes of RB69 pol that contained a correct incoming dNTP and a dideoxy-terminated primer/ template with a resolution of 2.6 Å and later at 1.8 Å were reported (Franklin et al. 2001; Wang et al. 2011). For many years, RB69 DNA polymerase became a prototype DNA polymerase that enabled structure fidelity studies for this class of polymerase. As a result of these intensive studies, nearly 170 independently observed structures of RB69 DNA polymerase have been deposited in the Protein Data Bank (PDB) (Ren 2016). RB69 DNA polymerase like T4 DNA polymerase is a processive and high fidelity enzyme responsible for coordinated replication of both leading and lagging DNA strands. Recently crystal structures for catalytic subunits of eukaryotic replicative polymerase δ, ɛ, and α have been obtained (Hogg et al. 2014; Perera et al. 2013; Swan et al. 2009).

Despite the amino-sequence differences, all replicative polymerase structures share a common overall architecture and are composed of five subdomains: N-terminal domain (NTD), exonuclease domain (exo), and polymerase domain (pol), the core of the enzyme. The polymerase domain contains a palm domain with several of the catalytic residues, a finger domain with most of the side chains that bind the incoming dNTP, and a thumb domain that binds primer-duplex DNA. The exonuclease domain carries a 3′-5′ proofreading activity that removes misincorporated nucleotides. The function of the N-terminal domain is not well defined. In RB69 and T4 polymerases, the NTD domain binds its messenger RNA and represses translation (Petrov et al. 2002). It was also shown that NTD could play a role in polymerase stability and fidelity through the interaction with the fingers domain (Li et al. 2010; Prindle et al. 2013). Pol δ NTD domain contains three motifs. One of these motifs has the structure and topology resembling an OB-fold which may be involved in single-stranded DNA binding. Other motifs contain an RNA-binding motif (RRM) that may be involved in RNA binding (Swan et al. 2009).

### A family polymerase structure

**Fig. 1:**
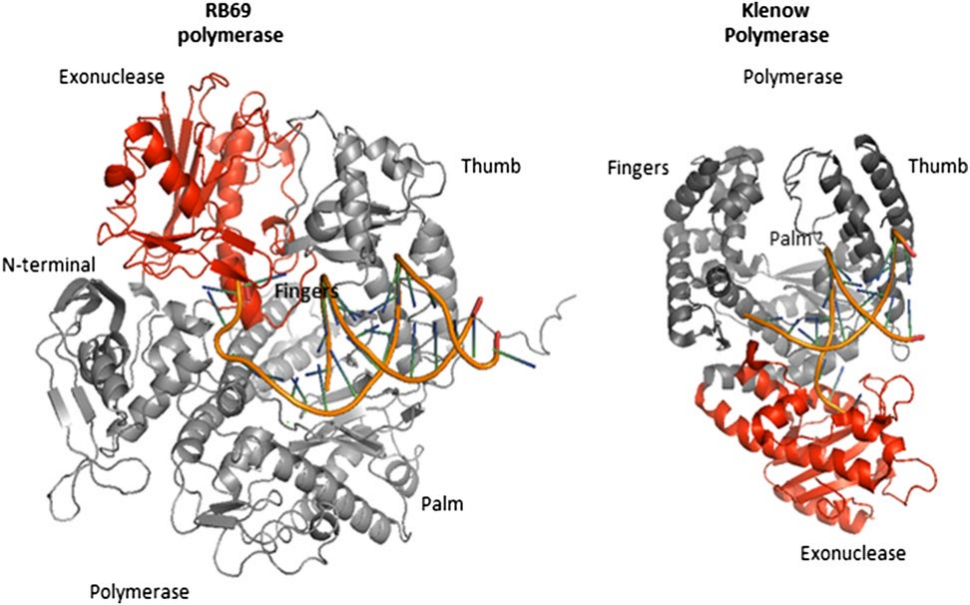
Position of the exonuclease and polymerase active sites in A family (Klenow polymerase) and B family (RB69 polymerase). The enzymes are in cartoon representation with the polymerase domain in grey and exonuclease domain in red and DNA in orange. The images were generated using PyMol (DeLano 2002), and are based on the crystal structure of Klenow in the complex with DNA (PDB ID code 1KLN) and the ternary complex structure of RB69 polymerase (PDB ID code 3NCI)

The pol A family polymerases, also known as the pol I family, have two separate enzymatic activities as well, a 5′-3′ polymerase activity and a 3′-5′ exonuclease activity on the same peptide (Klenow fragment). The polymerase domain of DNA pol I family resembles in overall the topology of the B family polymerase domain with fingers that bind an incoming nucleotide and interact with ssDNA, palm, which harbors catalytic residues, and thumb domain which binds ds DNA (Patel et al. 2001; Steitz 1999). The structures of the fingers and thumb domains are unique in A and B families. The palm domain structure shares the same fold in A and B family. The exonuclease domain is distal to the palm domain (Fig. 1) a location much different compared to B family (Kunkel and Bebenek 2000).

### C family polymerase structure

Like all polymerases, *E. coli* Pol III α contains finger, thumb, and palm domains. In contrast to A and B family, the palm domain structure of Pol III has the basic fold of Pol β like nucleotidyltransferase superfamily (X family). The palm domain contains three conserved catalytic aspartates that bind the catalytic magnesium ions and utilize the same two-metal ion catalytic mechanism as other polymerases (Lamers et al. 2006). Pol III has an additional domain PHP domain. PHP domain of Pol III has been proposed to act as a pyrophosphatase that hydrolyzes the pyrophosphate by-product of DNA synthesis (Aravind and Koonin 1998; Lamers et al. 2006). PHP domain from *Thermus aquaticus* Pol III was shown to have a 3′–5′ exonuclease that is Zn^2+^ dependent and may act as a second exonuclease (Stano et al. 2006). The proofreading domain is carried on a separate polypeptide (*dnaQ*) but is tightly associated with polymerase during DNA replication (Scheuermann and Echols 1984; Toste Rêgo et al. 2013).

### Polymerase active site

A detailed examination of the binary and ternary complex crystal structures of the pol I family of DNA polymerases has revealed that template-primer binding is associated with translational and rotational changes in the thumb subdomain, described as “clamping down” over DNA. Subsequently, dNTP binding induces movement in the fingers domain (mainly O-helix) by ∼ 41°, which in turn forms the “closed” ternary complex (Beese et al. 1993).

**Fig. 2:**
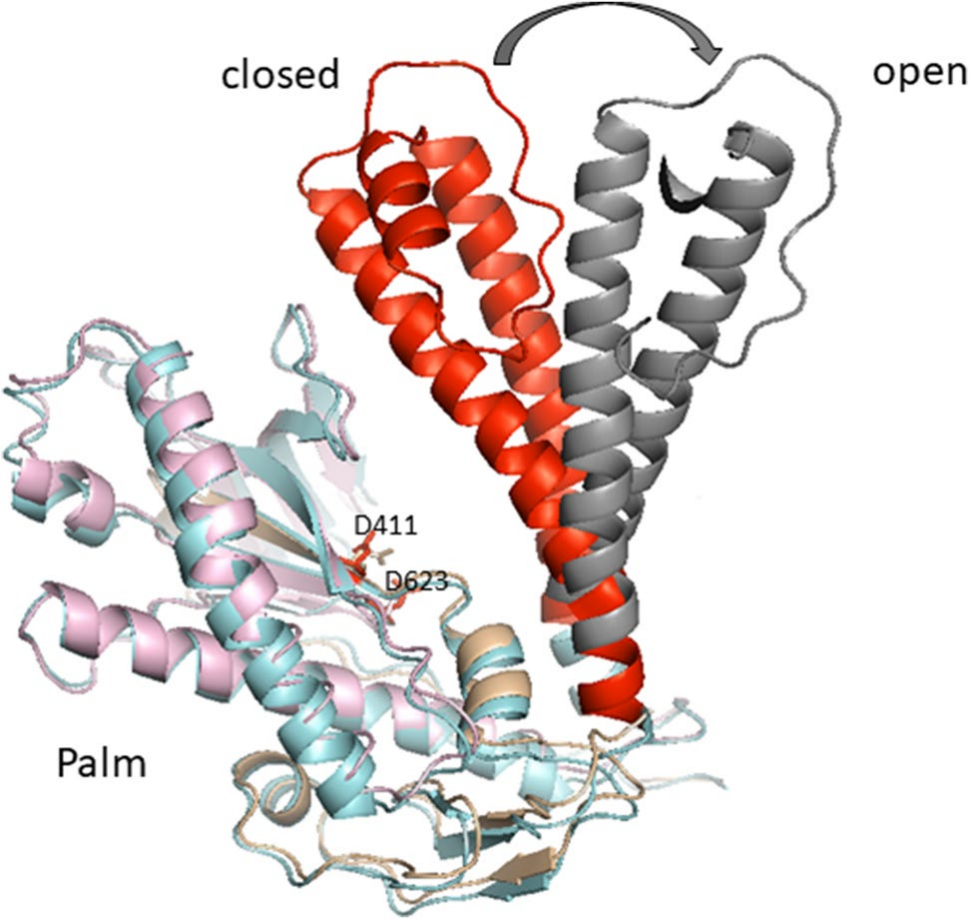
Movement of fingers domain from the “closed” to “open” conformation in RB69 DNA polymerase. Fingers’ movements brings conserved residues from motif B closer to palm catalytic residues D411 and D623 creating polymerase active site. The image was created using PyMol (DeLano 2002) and the ternary complex structure of RB69 polymerase (PDB ID code 3NCI)

The crystal structure of an RB69 gp43 ternary complex showed that binding of dNTP induces conformational changes in the polymerase from an open state in the absence of nucleotide to the closed state with the nucleotide. After binding of dNTP to the template primer, the fingers rotate about 60° towards the palm domain, bringing conserved residues from motif B closer to palm catalytic residues (Fig. 2). The palm subdomain harbors the catalytic core responsible for pol activity. Two highly conserved acidic residues (D411 and D623) serve as ligands for metal ions A and B, which are crucial for catalyzing the nucleotidyl transfer reaction. Two-metal Mg^2+^ ions are required to coordinate transition state in the nucleotide transfer reaction and assist in the departure of the PPi product (Xia and Konigsberg 2014). This two-metal requirement for catalysis seems to be general for all polymerases (Johnson 2010). However, recently, the possibility that a third metal ion can be involved in the catalysis of pol β and coordinate the PPi departure was postulated by (Yang et al. 2016).

Fingers’ domain contains a conserved dNTP-binding motif KX3NSXTG. Each phosphate group of the incoming dNTP is coordinated to a protein side chain. Both fingers and palm domain form a tight-binding pocket around the nascent base pair. The geometry of this pocket accommodates only a correct base pair with proper Watson–Crick geometry. The proper alignment of the incoming nucleotide with the templating nucleotide promotes catalysis and extension. Following the insertion of a correct nucleotide, polymerase must translocate to allow binding of the next nucleotide. If the mismatched nucleotide is bound, catalysis is slowed, and primer terminus is directed to the exonuclease active site (Xia and Konigsberg 2014).

Structural analysis of the related B family polymerases also revealed conformational changes after nucleotide binding from an open to closed state. Thus, finger “open” to “close” state during polymerization is a universal mechanism for all polymerases.

### The exonuclease active site

In all B family of DNA, Pols exonuclease contains three conserved motifs, Exo I, Exo II, and Exo III. Exonuclease active site, like the polymerase active site, contains essential aspartate residues that bind the two Mg^+^ ions that are required for the hydrolysis reaction via a two-metal mechanism (Bernad et al. 1989).

The exonuclease (exo) subdomain lies between the N-terminal and thumb subdomains. In RB69 polymerase, the exo-and-pol active sites are separated by about 30–40 Å. When an incorrect dNTP is incorporated onto the 3′ terminus of the primer strand, the pol helps to switch the primer terminus from the pol to the exo site, facilitating cleavage of the 3′-terminal nucleotide residue. To reach exo active site, primer strand has to separate from the template along three nucleotides to place the primer 3′-end in the exonuclease active site for editing (Shamoo and Steitz 1999). DNA polymerases with proofreading ability can sense misincorporated nucleotides by contacting the minor groove of base pairs beyond the insertion site. The polymerase senses the geometry of the base pair through specific hydrogen bond acceptors at the pyrimidine O-2 and purine N-3 atoms. This geometry is lost when the mismatches are present. RB69 DNA polymerase can sense the mismatches up to the two base pairs post the insertion site (Wang et al. 2011). These contacts are much more extensive in eukaryotic pol δ and extend to five base pairs (Doublie and Zahn 2014; Swan et al. 2009). In Pol A family, the active sites for the polymerase and exonuclease domains are also located in separate structural domains and are separated by about 30 Å in Klenow fragment and 35 Å in T7 pol (Beese et al. 1993; Ollis et al. 1985). The primer must melt the last four base pairs at the 3′ terminus of a duplex DNA to reach exonuclease active site (Lam et al. 1998). The 3′-5′ exonuclease domains are located on opposite sides of the pol active sites (Fig. 1) what causes differences in the coordination between the two active sites among B- and A family enzymes (Kunkel and Bebenek 2000).

**Fig. 3:**
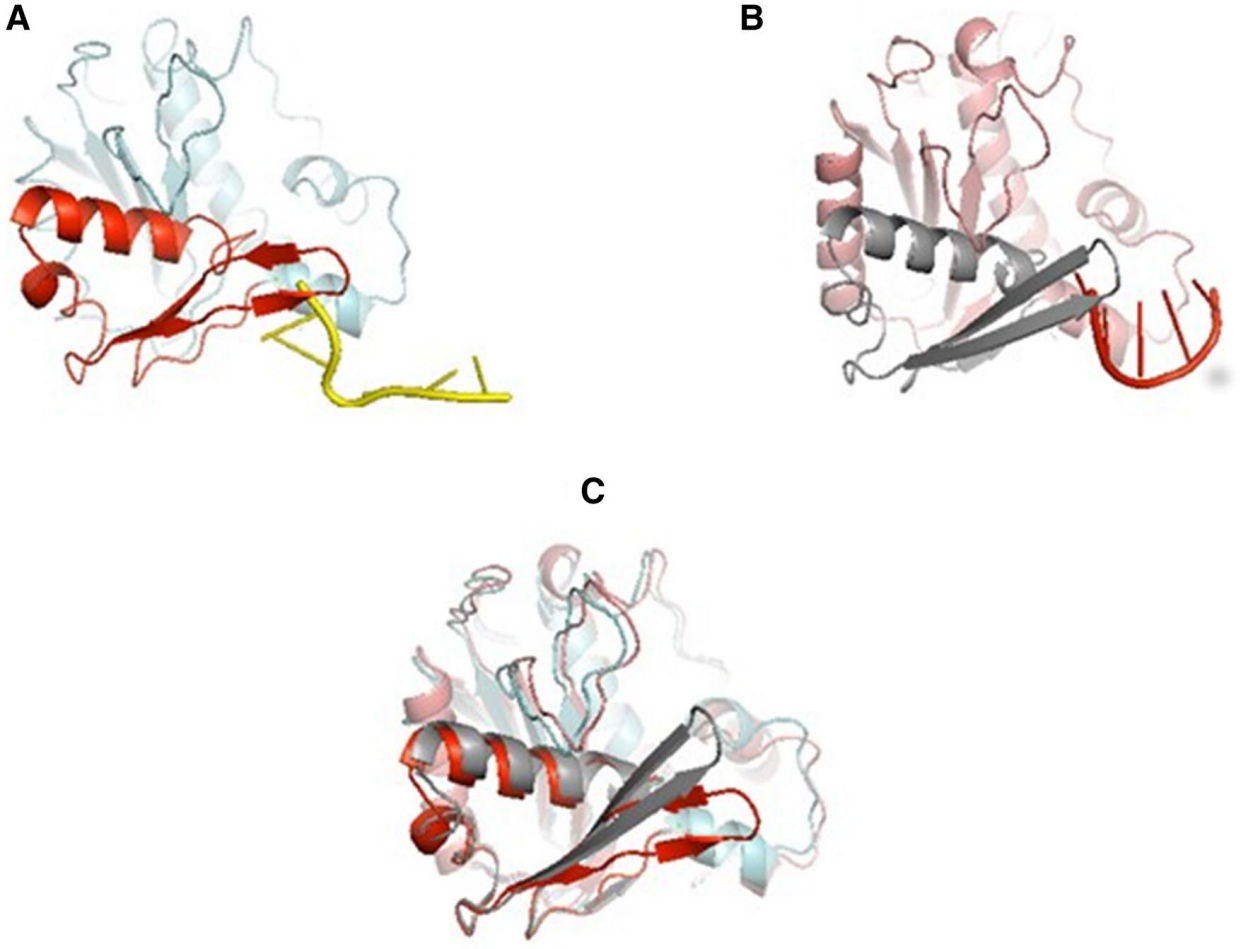
Position of the β-hairpin loop in editing (**a**) and replicating (**b**) modes. Superposition of the two structures showing the movement of the β-hairpin loop (**c**). The images were generated using PyMol (DeLano 2002) based on the ternary crystal structure of RB69 DNA polymerase (PDB ID code 3NCI) and editing structure (PDB ID 1CLQ)

In B family, the partitioning of the DNA primer between the polymerase and exonuclease active site is accompanied by the β-hairpin loop, a part of the exonuclease domain. In RB69 DNA polymerase, residues 251–262 form an extended hairpin loop (β-hairpin). In the editing mode, both DNA strands depart from the polymerase active site and β-hairpin loop holds the template strand in place, while the primer strand partially separates from the template strand and passes behind the β-hairpin to reach the exonuclease active site (Hogg et al. 2007; Ren 2016; Shamoo and Steitz 1999). Mutating residues in the loop of the β hairpin in T4 or RB69 DNA polymerases (G255S and G258S respectively) or deleting the loop of the β hairpin caused a mutator phenotype (Hogg et al. 2007; Trzemecka et al. 2009). Biochemical analysis showed that the mutant polymerases degraded ssDNA with the same efficiency as wild-type enzymes but have decreased ability to degrade dsDNA, thus showing that β hairpin loop is, indeed, essential in strand separation and does not affect exonuclease activity (Fig. 3) (Hogg et al. 2007; Subuddhi et al. 2008; Trzemecka et al. 2009). Almost all B family DNA polymerases whose structures have been solved to date show a similarly placed β-hairpin loop in the same orientation with respect to the polymerase and exonuclease active sites as in RB69 gp43. The β-hairpin is present in bacteriophage φ29 (Salas et al. 2008), herpes simplex virus (Liu et al. 2006), the archaeal polymerases (Hashimoto et al. 2001; Hopfner et al. 1999; Savino et al. 2004), pol II polymerase from *E. coli* (Wang and Yang 2009), and replicative eukaryotic polymerases δ and ɛ (Ganai et al. 2015; Swan et al. 2009). Although the β-hairpin loop is located in the same position within their exonuclease domain, not always play the same role as in RB69 gp43 and T4 gp43 (Table 1). DNA pol δ has similarly placed β-hairpin loop, but as it was demonstrated very recently, DNA pol δ does not need the hairpin for proofreading, but β-hairpin loop is required for optimum DNA replication efficiency, because its role is to stabilize polymerase complexes (Darmawan et al. 2015). The β-hairpin loop is truncated in pol ε, is too short to contact the DNA, and presumably is not involved in active site switching (Ganai et al. 2015). Instead, Pol ɛ has an additional large domain named the P domain that is built from insertions of residues 533–555 and 682–760 not previously observed in B family polymerases. The P domain is built of three β-strands, two α-helices, and a β-hairpin loop. Together, these structural motifs form an elongated domain that extends outward from the palm domain toward dsDNA. P domain allows the interactions between the pol ɛ and the duplex DNA and allows for sensing mismatches not only at the primer end but also at positions n-4 or n-5 that may destabilize the 3′-terminus of the primer at the polymerase active site and lead to a transfer of primer terminus to the exonuclease active site. The P domain may be an obvious replacement of β-hairpin loop, because it can help to maintain close contact between the polymerase and DNA while switching active sites. It can compensate the lack of β-hairpin loop (Ganai et al. 2015; Hogg et al. 2014).

**Table 1:** Function of β-hairpin loop in B family polymerases

Polymerase	β-hairpin loop	Role	Function
T4 and RB69	yes	Strand separation and positioning primer strand in exo site	Pol/exo coupling
*E. coli* pol II	Yes	Altered partitioning keeps the primer near pol active site	Translesion synthesis
Pol δ	Yes	Stabilize polymerase complexes	Optimum DNA replication efficiency
Pol ɛ	Yes but truncated	Not involved in strand separation	None

The *E. coli* Pol II that also participates in chromosomal DNA replication (Banach-Orlowska et al. 2005) has the 20 residue β barrel insertion in the N domain that shifts the position of the β-hairpin loop and alters the partitioning between polymerization and proofreading by keeping the primer end near the polymerase active site far from the exonuclease (Wang and Yang 2009). Because of these differences in β hairpin position, the Pol II can carry translesion synthesis past DNA lesion (Wang and Yang 2009).

## Pol-to-exo active site switching

Polymerases that have an exonuclease domain in the same polypeptide with polymerase domain can proofread errors intramolecularly, without enzyme dissociation from the mismatch primer terminus (Joyce 1989) or intermolecularly with enzyme dissociation and rebinding to the DNA to form exonuclease complexes (Johnson 1993; Reha-Krantz 2010).

The joint structural analysis of all known RB69 DNA polymerase structures enabled to extract structural changes during translocation of the polymerase along a DNA template and processive switching between the polymerase and exonuclease active sites (Ren 2016). Translocation of the polymerase is associated with the fingers motion from the closed after dNTP hydrolysis and release of PPi to an open conformation. When the correct Watson–Crick base pair is formed, the thumb domain is disengaged from the minor groove of the duplex DNA which is accompanied by overall rotation of the N-terminal and thumb domain around the DNA duplex and facilitates the relative sliding between protein and DNA. When the mismatch is formed, the thumb is constantly holding the duplex in the minor groove that avoids translocation and allows the primer to shuttle to and from the exonuclease active site. The thumb holds the DNA duplex in its minor groove with Lys734 and 800 (Ren 2016).

The intermolecular site switching which requires DNA polymerase dissociation was proposed for T4 DNA polymerase after it was observed that active T4 DNA polymerase exchange was taking place during T4 replisome replication in vitro (Yang et al. 2004). The T4 and RB69 DNA polymerases are not processive, and the processivity during replication is enhanced by the processivity factor, the product of gene *gp45* that forms a homotetrameric structure that encircles the DNA (Karam and Konigsberg 2000). Gp45 protein tether more than one T4 or RB69 polymerase and when the replicating polymerase dissociates from the mismatch primer end the same or another polymerase can rebind the mismatched DNA in the exonuclease active site (Yang et al. 2004). The intermolecular site switching can also operate for pol δ (Flood et al. 2015).

The intramolecular and intermolecular transfer of DNA between the pol and exo sites was recently also demonstrated for DNA polymerase I Klenow fragment by monitoring the movement of the DNA between these two active sites by a single-molecule Fӧrster resonance energy transfer (smFRET) method (Lamichhane et al. 2013).

### Switching between polymerase and exonuclease active sites can be modulated by the interaction with the sliding clamp

DNA sliding clamp assists most of the replicative polymerases in ensuring processive and accurate genome replication. Clamps despite their low level of sequence identity, from prokaryotes and eukaryotes, form a similar ring structure with a central hole that encircles duplex DNA. Polymerase interacts with PCNA by a small conserved PCNA-interacting protein motif (PIP-box). The PIP motif binds a hydrophobic patch on the PCNA surface. For archaeal DNA Pol B polymerase from *Pyrococcus furiosus* (Pfu Pol) based on computational analysis of all available structural information and molecular dynamics simulations, novel contacts were found between DNA polymerase and the PCNA subunits adjacent to PIP motif (Nishida et al. 2009; Xu et al. 2016). In the pol-mode, these interactions involve polymerase residue R706 (thumb domain) and residue E171 from PCNA1 subunit. In the exo-mode, interactions are made by a helix from palm domain that contains a patch of arginine residues (R379, R380, and R382) and a negatively charged loop on the PCNA2 subunit surface called the “switch hook” (Xu et al. 2016). It was proposed that the transition from the polymerase mode to exonuclease mode is executed by the rotation of the Pol core (palm, N-terminal, fingers, and exonuclease domain) around thumb domain that is stably bound to the clamp surface. It enforces a 56° rotation of a palm domain and brings the palm arginine patch helix into contact with the negatively charged switch-hook loop of PCNA2 that locks the complex in the exo-mode conformation. These results provided that PCNA can coordinate the transition between the pol and exo states during DNA replication (Xu et al. 2016).

In *E. coli*, it was shown that Pol III α interaction with a clamp is enhanced by the exonuclease that provides a second indirect interaction to the clamp. By doing so, it enhances the interaction between Pol III α and a clamp and provides the exonuclease with more efficient access to the DNA. Exonuclease binds to the clamp by a canonical clamp binding motif that is positioned immediately after the exonuclease catalytic domain. Exonuclease clamp interactions are required for optimal proofreading activity (Fernandez-Leiro et al. 2015; Park et al. 2018; Toste Rêgo et al. 2013).

### Modulation of pol and exo activity by the subunit composition in pol δ holoenzyme

The human pol δ is a heterotetramer consisting of the catalytic subunit p125 (POLD1) and three accessory subunits p50 (POLD2), p68 (POLD3), and p12 (POLD4) (Tahirov 2012). The p12 subunit is degraded in response to DNA damage converting Pol δ4 to Pol δ3. It was shown that Pol δ3 is less error prone due to greater proofreading ability and greater discrimination against mismatched primers and small lesions that are readily bypassed in a mutagenic manner by Polδ4 (Lee et al. 2017; Zhang et al. 2016).

### Proofreading in trans

The eukaryotic genome is replicated by three replicative polymerases, the pol α, pol δ, and pol ɛ. Polymerase α is responsible for the synthesis of the 20–30 nucleotides during Okazaki fragment initiation, that is further extended by lagging strand polymerase δ (Kunkel 2009; Kunkel et al. 1989). The fidelity of DNA pol α is low as this polymerase lacks its own proofreading (Kunkel et al. 1989). It is estimated that polymerase α contributes to the synthesis of about 1.5% of the eukaryotic genome, and with calculated base substitution error rate of 10^−4^, this polymerase would introduce many thousands of mismatches during each round of replication. It was shown that errors introduced by polymerase α are removed by the exonucleolytic proofreading of polymerase δ (Pavlov et al. 2006). Later, it was also demonstrated that polymerase δ could proofread errors introduced by polymerase ɛ acting in trans on the leading DNA strand, but polymerase ɛ was unable to correct Pol δ-dependent replication errors as well as errors made by Pol α. In addition, errors created by proofreading defective polymerase ɛ cannot be corrected by wild-type Pol ε polymerase (Flood et al. 2015; Pavlov et al. 2006).

### External 3′-5′ exonucleases

A number of DNA pol-unassociated 3′-5′ exonuclease have been identified in eukaryotic cells (Mason and Cox 2012). Some of these exonucleases have been observed to have the ability to remove 3′ mismatched termini from double-stranded DNA and thus to correct replication errors. Werner protein (WRN) belongs to the RecQ family of helicases (Gray et al. 1997). *WRN* encodes a 3′-5′ helicase and also a 3′-5′ exonuclease (Kamath-Loeb et al. 1998). It was shown that polymerase delta proofreading could be enhanced by the Werner protein. It was presented that WRN was able to proofread for Pol δ by removing 3′-terminal mismatches to enable primer extension by Pol δ. Consistent with this in vitro observations, it was demonstrated that WRN contributes to the maintenance of DNA synthesis fidelity in vivo. Cells expressing limiting amounts (~ 10% of normal) of WRN have elevated mutation frequencies compared with wild-type cells (Kamath-Loeb et al. 2012).

The tumor suppressor protein p53 also possess 3′-5′ exonuclease activity (Mummenbrauer et al. 1996) and can remove mismatches from replicating DNA strand (Huang 1998). It was shown in vitro that p53 protein enhances the replication fidelity of error-prone polymerase α (Hollstein et al. 1996). Later, it was demonstrated that polymerase α-primase (prim-pol) could form a complex with p53 in vivo. The purified prim-pol/p53 complex in vitro showed both exonuclease and polymerase activity (Melle and Nasheuer 2002) and was able to extend a mismatched DNA primer terminus. These data provided evidence that p53 can correct DNA replication error introduced by pol α.

## Contribution of proofreading to fidelity

The proofreading on average improves replication fidelity by about 10–1000-fold. The errors that escape proofreading are repaired later by the mismatch repair (MMR) (Kunkel 2009; McCulloch and Kunkel 2008). The contribution of the proofreading or the mismatch repair system can be directly measured in vivo by comparing spontaneous mutation rates in wild strain or in the strains that are defective in one of the correction pathways or both. Different types of errors are produced by the polymerase. The most common mistakes are base substitution errors. There are two types of base substitution errors, transitions (purine–purine and pyrimidine–pyrimidine mismatches) and transversions (purine-pyrimidine mismatches). The base substitution errors depend on the selectivity of the polymerase. Polymerase active site can bind some forms of these mispairs as they can adopt wobble conformation or exist in a rare tautomeric form (Goodman et al. 1993; Kunkel and Bebenek 2000). From the in vivo and in vitro fidelity measurements, it became evident that the transition mismatches are less efficiently proofread than transversion mismatches and are more easily extended by the polymerases. This unbiased preference for transition mismatches is corrected by mismatch repair system that discriminates more efficiently against transitions that transversions which at the end results at the same low level of both types of errors (Schaaper 1993). Another type of mutations that are introduced by replicative polymerases is frameshift mutations both deletion and addition errors (indels), especially in mononucleotide microsatellites. Microsatellites are tandem repeats of 1–6 base pairs per repeat unit that are found in all organisms, at varying abundance (Baptiste et al. 2015; Bebenek and Kunkel 2000; Kunkel and Bebenek 2000). The primer-template slippage during replication of repetitive sequences produces misaligned intermediates that are stabilized by a correct base and subsequent polymerization leads to deletion if the flipped nucleotide is in the template strand or to addition if the flipped nucleotide is in the primer strand (reviewed in Bebenek and Kunkel 2000). Frameshift mutations can also be generated at noniterated or short repetitive sequences. Proofreading can remove indel mutations in a short repetitive sequence and noniterated sequences with almost the same efficiency as base substitution mutations but is much less efficient in removing frameshift mutations in homopolymeric runs. These mutations are removed efficiently by mismatch repair system (Kunkel and Bebenek 2000; Yamamoto and Imai 2015). Instability of the microsatellite sequences is associated with many disease states including cancer (Yamamoto and Imai 2015).

### Replicative polymerases in cancer

Studies in the model organisms have confirmed the essential role of DNA polymerase proofreading in the maintenance of genomic stability. Exonuclease-deficient mutants of Pol δ or Pol ɛ containing alanine substitution at catalytic aspartate residue in *S. cerevisiae* show a 10–100-fold increase in mutation rates (Morrison et al. 1993; Pavlov et al. 2001; Shcherbakova et al. 2003b). In the mice model, when the exonuclease domain of Pol δ (encoded by the *POLD1* gene) or Pol ɛ (encoded by the *POLE* gene) was inactivated by mutation at exonuclease, catalytic residue elevated base substitution mutation rates, and increased incidence of cancers was observed. The type of cancers was different for each polymerase mutant. Pol δ exo- mice developed lymphomas and carcinomas of the skin and lung, whereas Pol ɛ exo- mice developed intestinal tumors (Albertson et al. 2009; Goldsby et al. 2002, 2001). Interestingly, cancers only developed in mice homozygous for proofreading deficient *polD1* and *polE* alleles and in mismatch proficient background (Goldsby et al. 2002). That was the first such example showing that mutations in genes encoding polymerases could be a source for multiple mutations that if accumulated over the lifetime can increase the risk of cancer.

In 2012, The Cancer Genome Atlas (TCGA) (Network 2012) published the results of analysis of exome sequencing of 224 sporadic colorectal carcinomas (CRC). These study revealed that a subset of ultramutated but microsatellite stable (MSS) CRC tumors with the highest mutational load had alterations in *POLE* gene. *POLE* alterations were also found in hypermutated sporadic endometrial tumors (Church et al. 2013).

The changes were found within and close to the Exo motifs required for exonuclease activity, suggesting that inactivation of exonuclease activity was responsible for the hypermutator phenotype (Rayner et al. 2016). The hypermutator phenotype observed in *POLE* proofreading domain mutant is characterized by an excess of substitution mutations, in particular, a relative excess of G:C→T:A transversions (Alexandrov et al. 2013). The most common *POLE* variant is the replacement of the proline 286 by either the arginine or histidine. The functional consequences of P286R mutation were studied using a yeast homolog *pol2*-P301R mutant. The corresponding P301R change in yeast Pol ε conferred an exceptionally strong mutator phenotype greatly exceeding that of any previously characterized Pol ε mutant, including proofreading-deficient mutants. It was also shown that heterozygosity for the P301R also produced a strong mutator effect comparable with that of MMR deficiency, the effect not observed for proofreading-deficient pol2 exo-polymerase (Kane and Shcherbakova 2014).

Only two cases of possibly pathogenic somatic *POLD1* variation have been identified to date (Shlien et al. 2015).

In addition, germline mutations affecting the exonuclease domains of *POLE* and *POLD1* were found to cause a high-penetrance hereditary colorectal cancer and endometrial cancer predispositions (Bellido et al. 2016). These discoveries strongly suggested that loss of proofreading activity of replicative DNA polymerases is the initiating cause of some hereditary and sporadic human cancers.

The role of proofreading domain mutations in cancer has been recently extensively reviewed (Barbari and Shcherbakova 2017; Rayner et al. 2016).

## Concluding remarks

Replicative polymerases use several mechanisms to achieve high and accurate DNA replication. Proofreading plays an essential role in this process. Proofreading activity either is associated with the polymerase or carried on a separate subunit, but in any case, it is estimated that proofreading improves replication fidelity by a factor of 10^2^–10^3^. Recent studies with eukaryotic replicative polymerases that have been found in some cancers showed that mutations in exonuclease domain close to catalytic residues could cause much stronger mutator phenotype exceeding the previously observed for exo-deficient polymerases (Kane and Shcherbakova 2014). A mechanism that would explain such high level of mutagenesis is not known.

The interplay between the pol and exo activity may be modulated by the interaction with one of the accessory proteins that accompany polymerase during DNA replication, like interactions with β sliding clamp and ε subunit in *E. coli* replisome (Park et al. 2018; Xu et al. 2016). It was shown that mutations in a non-catalytic subunit of Pol ɛ, Dpb2, that destabilize interactions with Psf1 and Psf3 subunits in GINS complex result in increased spontaneous mutagenesis in yeast *S. cerevisiae* (Dmowski and Fijałkowska 2017; Garbacz et al. 2015). One can speculate that mutations in the exonuclease domain that does not affect catalytic site may affect primer terminus site switching between pol and exo site, sending more often uncorrected primer back to polymerases active site or not allowing the primer terminus to reach the exo active site. Many other scenarios can take place, and it is a long way to fully understand the mechanism that is responsible for the coordinated action of polymerase and exonuclease activity. Finally, it can be assumed that the proofreading may participate too much higher extent in replication fidelity that it was previously anticipated.
